# Phlegmonous Ileocolitis as a Presentation of Post SARS-CoV-2 (COVID-19) Multisystem Inflammatory Syndrome in Children

**DOI:** 10.1097/PG9.0000000000000040

**Published:** 2020-12-17

**Authors:** Abdul R. Shahein, Heather Young, Abdallah Dalabih

**Affiliations:** From the * Division of Pediatric Gastroenterology; † Division of Pediatric Infectious Disease; ‡ Division of Pediatric Critical Care, Arkansas Children’s Hospital, University of Arkansas for Medical Sciences, Little Rock, AR.

Phlegmonous inflammation of the alimentary tract is characterized by diffuse inflammatory infiltrates of the submucosa of a portion of the intestine. The inflammatory reaction could reach the serosal surface with subsequent development of peritonitis or septicemia (1). SARS-CoV-2 is a novel virus responsible for COVID-19 that is causing a global pandemic causing mortality already in over half a million people around the world. In May 2020, the Center for Disease Control released a case definition for the newly described multisystem inflammatory syndrome in children (MIS-C) believed to be precipitated by COVID-19 that includes an individual aged <21 years presenting with fever ≥38.0°C for at least 24 hours, laboratory evidence of inflammation, evidence of clinically severe illness requiring hospitalization, and multisystem (≥2) organ involvement (cardiac, renal, respiratory, hematologic, gastrointestinal, dermatologic, or neurological); AND no plausible alternative diagnoses; AND positive for current or recent SARS-CoV-2 infection by real-time polymerase chain reaction (PCR), serology, or antigen test, or exposure to a suspected or confirmed COVID-19 case within the 4 weeks before the onset of symptoms. MIS-Cs pathophysiology remains unclear. However, it appears to be a postinfectious hyperimmune response that may occur during or following asymptomatic or symptomatic infection (2).

## CASE PRESENTATION

A 6-year-old male, undervaccinated and with a history of exposure to COVID-19, presented with acute onset high-grade fever, nonbilious nonbloody vomiting, periumbilical diffuse abdominal pain, and watery diarrhea. Patient symptoms worsened over the prior 7 days. His caregiver was diagnosed with symptomatic COVID-19 infection by PCR about 6–8 weeks before presentation. However, the patient had no symptoms and was not tested at that time. He had no history of gastrointestinal disease or weight loss prior to this illness.

**FIGURE 1.**
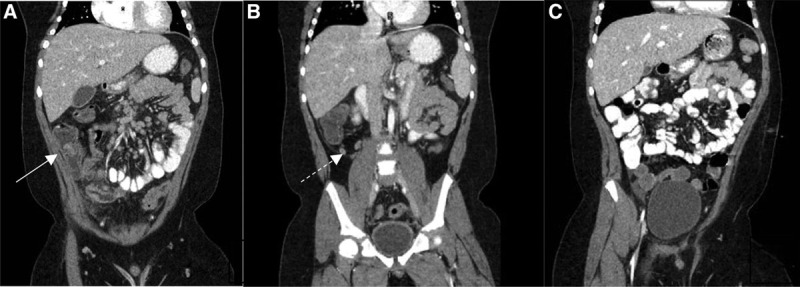
CT scan with oral and intravenous contrast of the abdomen and pelvis. A and B, CT scan of the abdomen and pelvis on presentation showing extensive phlegmonous changes in the right lower quadrant of the abdomen around the ileocecal junction (white arrowhead) with intact appendiceal wall (dashed arrowhead). C, CT scan of the abdomen and pelvis demonstrating complete resolution of phlegmonous ileocolitis after treatment. CT = computed tomography.

On presentation, he complained of shortness of breath and fatigue. Physical examination revealed fever (104°F) and toxic appearance. He had tachycardia with a short self-resolved episode of heart rate over 200 beats/min and had muffled heart sounds. He showed tachypnea, intercoastal retraction, and diffuse abdominal tenderness without peritoneal signs. Computerized tomography (CT) scan of the abdomen and pelvis with contrast showed extensive phlegmonous changes in the right lower quadrant around the ileocecal junction and terminal ileal loops. The terminal ileal loops, cecum, and ascending colon had wall thickening and abnormal enhancement. The ileocecal valve was edematous and was protruding into the cecum. Multiple enlarged (up to 2 cm) reactive lymph nodes were seen at the base of the mesentery and right lower quadrant. The CT scan also showed retrocolonic positioning of the appendix with borderline wall thickness and minimal fat stranding around the appendix (Fig. 1A, B).

Blood work revealed evidence of inflammation including elevated C-reactive protein, erythrocyte sedimentation rate, fibrinogen, procalcitonin, d-dimer, ferritin, and neutrophils, and reduced lymphocytes and albumin. Transthoracic echocardiogram showed mildly dilated left and right atrium, trivial mitral and tricuspid valve insufficiency, and moderately diminished left and right ventricular systolic function. The estimated left ventricular ejection fraction by volume was 33% with normal diameter coronaries. Chest X-ray was positive for pulmonary edema and signs of fluid overload. Infectious evaluation was positive for COVID-19 immunoglobulin G with negative nasopharyngeal PCR (Table 1).

**TABLE 1.** Summary of work up completed on admission

Test	Result	Normal range
Total leucocytic count (K/μl)	8.1	5–14.5
Hemoglobin (K/μl)	11.1*	11.5–15.5
Hematocrit	34.9%*	35–45%
Platelet count (K/μl)	160	150–400
Neutrophil count (K/μl)	6.81 (89%)	1.5–8.0
Lymphocytic count (K/μl)	0.89 (11%)*	1.5–7.0
Serum albumin	2.5 mg/dl*	3.5–5.2 g/dl
Aspartate aminotransferase (U/L)	38	15–50
Alanine transaminase (U/L)	44*	10–25
CRP (mg/L)	>88*	<4.0
Procalcitonin (ng/ml)	3.84*	<0.5
Ferritin (μg/L)	705*	20–310
Fibrinogen (mg/dl)	532.2*	150–400
D-dimers (mg/L)	7.04*	0.17–0.59
Heart-specific troponin I (pg/ml)	740.16*	<45.00
Beta naturetic peptide (pg/ml)	934.11*	<2.00–80.00
Urine protein	100 mg/dl (trace)	Negative to trace
Infectious work up		
Blood culture	No growth	
Urine culture	No growth	
Stool culture	No growth	
Cryptosporidium stool antigen	Negative	
Giardia stool antigen	Negative	
Clostridia difficile stool PCR	Negative	
Epstein Barr virus blood PCR	Negative	
Respiratory panel PCR nasopharynx†	Negative	
COVID-19 PCR nasopharynx	Negative	
COVID-19 Immunoglobulin G	Positive*	

*Abnormal value.

†Panel including polymerase chain reaction testing for the following organisms: adenovirus, Coronavirus Hong Kong University 1, coronavirus Netherland 63, human metapneumovirus, Flu virus A, B, H1N1, AH1 and AH3, parainfluenza virus 1, 2, 3, and 4, respiratory synthetical virus, Coronavirus 229E, Coronavirus OC34, *Bordetella pertussis*, *Bordetella parapertussis*, *Chlamydophila pneumonia*, and *Mycoplasma pneumonia*.

CRP = C-reactive protein; PCR = polymerase chain reaction.

Our patient was treated with intravenous ceftriaxone and metronidazole, immunoglobulin (IVIG) (2 g/kg/dose) for 2 doses within 24 hours, and methylprednisolone (1 mg/kg/dose twice daily for 3 days). He required inotropic support with milrinone. After initiation of IVIG and steroids, the patient showed improvement in fever and other systemic symptoms, including abdominal pain and diarrhea. Repeat CT scan of the abdomen and pelvis with contrast after 7 days of therapy showed complete disappearance of the previously found ileocecal phlegmon (Fig. 1C).

## DISCUSSION

Our patient was diagnosed with MIS-C based on the Center for Disease Control’s recently released case definition. Although he did not report symptoms of COVID-19 infection before the acute presentation with myocarditis and phlegmonous ileocolitis, he had a history of exposure and a positive serum COVID-19 Immunoglobulin G.

Phlegmonous inflammation of the alimentary tract is a unique presentation of the novel SARS-CoV-2 virus. Historically, this inflammation is hypothesized to be of bacterial origin, but its portal of entry and the pathogenesis remains unclear. Phlegmon is often seen with Crohn’s disease of the intestine. However, our patient did not report chronic gastrointestinal symptoms, and stool studies showed white blood cells count within normal limits. Mimics of inflammatory bowel disease (IBD) include not only infectious causes of colitis, but also vascular diseases, radiation-related injuries, drug-induced inflammation, and monogenic disorders in very-early-onset refractory IBD. A superinfection with cytomegalovirus or *Clostridium difficile* can aggravate intestinal inflammation in IBD, especially in patients who are immunocompromised (4,5).

Our patient had a negative comprehensive infectious evaluation (Table 1), except for positive COVID-19 IgG which indicates a previous infection that occurred prior to presentation.

In conclusion, this is the first reported case of phlegmonous ileocolitis secondary to initially asymptomatic COVID-19 infection. It is prudent for physicians including gastroenterologists during the COVID-19 era to be aware of this unique presentation of the novel virus to avoid unnecessary or invasive surgical intervention.

## References

[1] Gerard PW Phlegmonous colitis. Report of a case. Am J Clin Pathol. 1969;51:338–343. 4885297 10.1093/ajcp/51.3.338

[2] Feldstein LR Rose EB Horwitz SM; Overcoming COVID-19 Investigators; CDC COVID-19 Response Team. Multisystem Inflammatory Syndrome in U.S. Children and Adolescents. N Engl J Med. 2020;383:334–346. 32598831 10.1056/NEJMoa2021680 PMC7346765

[3] Shepherd NA Pathological mimics of chronic inflammatory bowel disease. J Clin Pathol. 1991;44:726–733. 1918397 10.1136/jcp.44.9.726 PMC496717

[4] Gecse KB Vermeire S Differential diagnosis of inflammatory bowel disease: imitations and complications. Lancet Gastroenterol Hepatol. 2018;3:644–653. 30102183 10.1016/S2468-1253(18)30159-6

[5] Rowley AH Understanding SARS-CoV-2-related multisystem inflammatory syndrome in children. Nat Rev Immunol. 2020;20:453–454. 32546853 10.1038/s41577-020-0367-5 PMC7296515

